# Supplementary material for: CognoStroke: Automated Cognitive and Mood Assessment on the Hyper-Acute Stroke Unit
Source: Healthcare (Basel). 2025 Nov 13;13(22):2885. doi: 10.3390/healthcare13222885 (PMC12652918; doi:10.3390/healthcare13222885)
Supplement: Supplementary file 1 [file healthcare-13-02885-s001.zip › healthcare-3914281-supplementary.pdf]

Supplementary Materials. Automated Language Processing

Further analysis of the LLM classification is provided in the Supplementary Tables S1–S3 below. Since the number of below and above thresholds are not equal, we have an imbalanced distribution of classes. So, we needed to consider averaging measures. Macro precision (MPR) and Macro recall (MRC) refers to averaging individual classes with an equal weight. The figure for the confusion matrix (CM) is also displayed to show how many of each individual classes are recognised correctly and how many are mixed with the other classes. Another way to think of the confusion matrix is displaying the true positives, false positives, false negatives, and true negatives in this order. An ideal classifier should assign all participants in a model into either the true positive of true negatives group. In the below tables the CM is displayed [true positives; false positives]; [false negatives; true negatives].

Supplementary Table S1: LLM performance on all prompts.

Outcome Measure	THR	Model	MPR	MRC	MFS	SP	SN	AUC	ACC	PPV	NPV	CM
MoCA	26	BART	0.758 [0.660–0.850]	0.741 [0.644–0.833]	0.720 [0.608–0.824]	0.903 [0.786–1.000]	0.579 [0.421–0.732]	0.741 [0.644–0.833]	0.725 [0.623–0.826]	0.880 [0.737–1.000]	0.636 [0.488–0.775]	[[28;3]; [16;22]]
	25	GPT2	0.706 [0.591–0.815]	0.697 [0.586–0.804]	0.696 [0.580–0.804]	0.795 [0.658–0.915]	0.599 [0.417–0.769]	0.697 [0.586–0.804]	0.710 [0.594–0.812]	0.692 [0.500–0.864]	0.720 [0.581–0.850]	[[31;8]; [12;18]]
	24	RoB-ERTa	0.640 [0.523–0.754]	0.659 [0.528–0.785]	0.641 [0.517–0.758]	0.740 [0.611–0.855]	0.579 [0.350–0.800]	0.659 [0.528–0.785]	0.695 [0.580–0.797]	0.457 [0.250–0.654]	0.822 [0.702–0.929]	[[37;13]; [8;11]]
	23	GPT2	0.761 [0.530–0.948]	0.636 [0.514–0.777]	0.657 [0.497–0.813]	0.964 [0.909–1.000]	0.308 [0.077–0.583]	0.636 [0.514–0.777]	0.841 [0.754–0.928]	Nan [nan–nan]	0.858 [0.766–0.938]	[[54;2]; [9;4]]
	22	GPT2	0.750 [0.413–0.962]	0.582 [0.483–0.714]	0.595 [0.448–0.765]	0.983 [0.945–1.000]	0.182 [0.000–0.444]	0.582 [0.483–0.714]	0.855 [0.768–0.928]	Nan [nan–nan]	0.864 [0.776–0.940]	[[57;1]; [9;2]]
PHQ-9	10	BART	0.695 [0.559–0.827]	0.680 [0.552–0.808]	0.682 [0.550–0.804]	0.499 [0.263–0.733]	0.860 [0.755–0.945]	0.680 [0.552–0.808]	0.765 [0.662–0.868]	0.827 [0.717–0.923]	0.564 [0.308–0.812]	[[9;9]; [7;43]]
	5	BART	0.647 [0.531–0.762]	0.647 [0.531–0.763]	0.644 [0.529–0.760]	0.648 [0.484–0.806]	0.647 [0.484–0.806]	0.647 [0.531–0.763]	0.647 [0.529–0.765]	0.647 [0.484–0.806]	0.647 [0.484–0.806]	[[22;12]; [12;22]]
GAD-7	10	RoB-ERTa	0.404 [0.353–0.449]	0.500 [0.500–0.500]	0.447 [0.414–0.473]	0.000 [0.000–0.000]	1.000 [1.000–1.000]	0.500 [0.500–0.500]	0.809 [0.706–0.897]	0.809 [0.706–0.897]	Nan [nan–nan]	[[0;13]; [0;55]]
	5	BART	0.630 [0.515–0.742]	0.632 [0.515–0.746]	0.614 [0.498–0.732]	0.715 [0.538–0.875]	0.550 [0.395–0.705]	0.632 [0.515–0.746]	0.618 [0.500–0.735]	0.734 [0.567–0.889]	0.526 [0.364–0.686]	[[20;8]; [18;22]]

Binary classification results with different thresholds (**THR**) on features extracted from all prompts, in terms of Macro F1-score (**MFS**) (the main metric), Specificity (**SP**), Sensitivity (**SN**), and area under the curve (**AUC**) using the three text-based foundation models, **GPT2**, Facebook.BART-base (**BART**), and RoBERTA-base (**RoBERTA**). Macro precision (**MPR**) and Macro recall (**MRC**) refers to averaging individual classes with an equal weight. Positive predicted value **(PPV**) and negative predicted values **(NPV**) are displayed. The figure for the confusion matrix (**CM**) is also displayed. Numbers in square brackets for each statistical feature represent the 95% confidence interval for that measure. **Nan** indicates when a value cannot be computed due to the structure of the confusion matrix.

Supplementary Table S2: LLM performance on single prompts.

Outcome Measure	THR	Model	MPR	MRC	MFS	SP	SN	AUC	ACC	PPV	NPV	CM
MoCA	26	RoB-ERTa	0.693 [0.580–0.801]	0.694 [0.581–0.804]	0.691 [0.578–0.797]	0.678 [0.500–0.840]	0.711 [0.561–0.848]	0.694 [0.581–0.804]	0.696 [0.580–0.797]	0.730 [0.578–0.868]	0.657 [0.484–0.818]	[[21;10]; [11;27]]
		GPT2	0.680 [0.566–0.789]	0.681 [0.567–0.792]	0.677 [0.563–0.783]	0.678 [0.500–0.840]	0.685 [0.531–0.829]	0.681 [0.567–0.792]	0.682 [0.565–0.797]	0.723 [0.567–0.865]	0.637 [0.467–0.800]	[[21;10]; [12;26]]
		GPT2	0.669 [0.558–0.779]	0.671 [0.558–0.782]	0.663 [0.549–0.776]	0.710 [0.545–0.867]	0.631 [0.474–0.784]	0.671 [0.558–0.782]	0.667 [0.551–0.783]	0.728 [0.567–0.875]	0.611 [0.444–0.769]	[[22;9];[14;24]]
	25	GPT2	0.693 [0.581–0.798]	0.696 [0.582–0.803]	0.690 [0.578–0.797]	0.692 [0.543–0.833]	0.700 [0.531–0.853]	0.696 [0.582–0.803]	0.695 [0.580–0.797]	0.636 [0.469–0.800]	0.750 [0.600–0.882]	[[27;12]; [9;21]]

		BART	0.690 [0.580–0.794]	0.691 [0.580–0.795]	0.678 [0.564–0.782]	0.615 [0.457–0.769]	0.766 [0.607–0.906]	0.691 [0.580–0.795]	0.681 [0.565–0.783]	0.605 [0.447–0.763]	0.774 [0.615–0.909]	[[24;15]; [7;23]]
		BART	0.660 [0.548–0.768]	0.662 [0.548–0.770]	0.649 [0.535–0.762]	0.590 [0.432–0.744]	0.733 [0.567–0.880]	0.662 [0.548–0.770]	0.652 [0.536–0.768]	0.579 [0.421–0.737]	0.742 [0.579–0.886]	[[23;16]; [8;22]]
	24	RoB-ERTa	0.746 [0.612–0.869]	0.729 [0.603–0.848]	0.732 [0.605–0.848]	0.879 [0.780–0.961]	0.579 [0.350–0.800]	0.729 [0.603–0.848]	0.797 [0.696–0.884]	0.646 [0.400–0.875]	0.846 [0.740–0.940]	[[44;6]; [8;11]]
		GPT2	0.711 [0.600–0.820]	0.748 [0.624–0.858]	0.716 [0.598–0.829]	0.759 [0.633–0.872]	0.737 [0.524–0.933]	0.748 [0.624–0.858]	0.753 [0.652–0.855]	0.537 [0.345–0.727]	0.884 [0.780–0.974]	[[38;12]; [5;14]]
		GPT2	0.684 [0.567–0.799]	0.705 [0.578–0.828]	0.687 [0.564–0.804]	0.780 [0.660–0.887]	0.631 [0.400–0.850]	0.705 [0.578–0.828]	0.739 [0.638–0.841]	0.520 [0.312–0.722]	0.848[0.735–0.947]	[[39;11]; [7;12]]
	23	BART	0.739 [0.584–0.888]	0.725 [0.579–0.869]	0.726 [0.579–0.859]	0.910 [0.828–0.981]	0.540 [0.250–0.818]	0.725 [0.579–0.869]	0.841 [0.754–0.928]	0.583 [0.286–0.875]	0.895 [0.808–0.966]	[[51;5]; [6;7]]

		BART	0.713 [0.550–0.873]	0.687 [0.541–0.835]	0.692 [0.542–0.832]	0.910[0.828–0.981]	0.463 [0.182–0.750]	0.687 [0.541–0.835]	0.826 [0.739–0.913]	0.545 [0.231–0.857]	0.880 [0.789–0.952]	[[51;5];[7;6]]
		BART	0.713 [0.550–0.873]	0.687 [0.541–0.835]	0.692 [0.542–0.832]	0.910 [0.828–0.981]	0.463 [0.182–0.750]	0.687 [0.541–0.835]	0.826 [0.739–0.913]	0.545 [0.231–0.857]	0.880 [0.789–0.952]	[[51;5]; [7;6]]
	22	RoB-ERTa	0.644 [0.529–0.763]	0.715 [0.550–0.868]	0.654 [0.517–0.786]	0.793 [0.683–0.891]	0.638 [0.333–0.909]	0.715 [0.550–0.868]	0.768 [0.667–0.870]	0.368 [0.150–0.593]	0.920 [0.837–0.981]	[[46;12]; [4;7]]
		RoB-ERTa	0.634 [0.524–0.751]	0.707 [0.542–0.862]	0.642 [0.507–0.771]	0.776 [0.661–0.877]	0.638 [0.333–0.909]	0.707 [0.542–0.862]	0.754 [0.652–0.855]	0.350 [0.143–0.571]	0.919 [0.833–0.981]	[[45;13]; [4;7]]
		RoB-ERTa	0.626 [0.533–0.728]	0.700 [0.559–0.838]	0.640 [0.530–0.752]	0.828 [0.752–0.900]	0.572 [0.300–0.833]	0.700 [0.559–0.838]	0.797 [0.717–0.867]	0.320 [0.143–0.517]	0.932 [0.875–0.978]	[[82;17]; [6;8]]
PHQ-9	10	BART	0.716 [0.584–0.843]	0.707 [0.579–0.831]	0.707 [0.580–0.827]	0.554 [0.312–0.786]	0.860 [0.755–0.945]	0.707 [0.579–0.831]	0.779 [0.676–0.868]	0.843 [0.736–0.938]	0.589 [0.348–0.824]	[[10;8]; [7;43]]

		GPT2	0.687 [0.567–0.807]	0.705 [0.574–0.829]	0.690 [0.564–0.808]	0.610[0.375–0.833]	0.800 [0.681–0.904]	0.705[0.574–0.829]	0.750 [0.647–0.853]	0.850 [0.739–0.941]	0.524 [0.304–0.739]	[[11;7]; [10;40]]
		BART	0.674 [0.555–0.791]	0.695 [0.563–0.820]	0.677 [0.551–0.796]	0.610 [0.375–0.833]	0.780 [0.660–0.889]	0.695 [0.563–0.820]	0.735 [0.632–0.838]	0.847 [0.735–0.941]	0.500 [0.286–0.714]	[[11;7]; [11;39]]
	5	RoB-ERTa	0.687 [0.569–0.798]	0.677 [0.563–0.782]	0.669 [0.549–0.779]	0.560 [0.390–0.727]	0.794 [0.649–0.923]	0.677 [0.563–0.782]	0.677 [0.559–0.779]	0.643 [0.500–0.784]	0.731 [0.545–0.895]	[[19;15]; [7;27]]
		BART	0.668 [0.550–0.780]	0.661 [0.549–0.772]	0.654 [0.539–0.765]	0.765 [0.618–0.900]	0.558 [0.389–0.725]	0.661 [0.549–0.772]	0.661 [0.544–0.779]	0.703 [0.520–0.870]	0.634 [0.485–0.780]	[[26;8]; [15;19]]
		BART	0.669 [0.551–0.780]	0.662 [0.549–0.771]	0.655 [0.537–0.765]	0.560 [0.390–0.727]	0.765 [0.613–0.900]	0.662 [0.549–0.771]	0.662 [0.544–0.779]	0.635 [0.487–0.780]	0.704 [0.520–0.867]	[[19;15]; [8;26]]
GAD-7	10	RoB-ERTa	0.702 [0.570–0.833]	0.735 [0.585–0.877]	0.710 [0.572–0.838]	0.616 [0.333–0.882]	0.854 [0.755–0.942]	0.735 [0.585–0.877]	0.809 [0.706–0.897]	0.904 [0.816–0.980]	0.500 [0.250–0.750]	[[8;5]; [8;47]]

		GPT2	0.621 [0.462–0.791]	0.598 [0.470–0.738]	0.601 [0.460–0.744]	0.306 [0.071–0.571]	0.891 [0.804–0.964]	0.598[0.470–0.738]	0.779 [0.676–0.868]	0.844 [0.745–0.931]	Nan [nan–nan]	[[4;9]; [6;49]]
		BART	0.597 [0.505–0.694]	0.628 [0.507–0.753]	0.601 [0.496–0.705]	0.472 [0.250–0.706]	0.784 [0.694–0.867]	0.628 [0.507–0.753]	0.729 [0.645–0.813]	0.873 [0.795–0.941]	0.321 [0.152–0.500]	[[9;10]; [19;69]]
	5	BART	0.650 [0.528–0.766]	0.649 [0.528–0.764]	0.646 [0.526–0.761]	0.572 [0.382–0.759]	0.725 [0.583–0.860]	0.649 [0.528–0.764]	0.662 [0.544–0.765]	0.708 [0.558–0.842]	0.593 [0.400–0.778]	[[16;12]; [11;29]]
		BART	0.618 [0.502–0.731]	0.622 [0.502–0.737]	0.611 [0.493–0.727]	0.644 [0.458–0.818]	0.600 [0.444–0.750]	0.622 [0.502–0.737]	0.618 [0.500–0.735]	0.706 [0.543–0.857]	0.530 [0.357–0.697]	[[18;10]; [16;24]]
		BART	0.654 [0.545–0.760]	0.616 [0.532–0.701]	0.608 [0.510–0.703]	0.377 [0.237–0.522]	0.855 [0.762–0.939]	0.616 [0.532–0.701]	0.654 [0.561–0.738]	0.654 [0.548–0.757]	0.654 [0.462–0.840]	[[17;28]; [9;53]]

Binary classification results with different thresholds (**THR**) on features extracted from all prompts, in terms of Macro F1-score (**MFS**) (the main metric), Specificity (**SP**), Sensitivity (**SN**), and area under the curve (**AUC**) using the three text-based foundation models, **GPT2**, Facebook.BART-base (**BART**), and RoBERTA-base (**RoBERTA**). Macro precision (**MPR**) and Macro recall (**MRC**) refers to averaging individual classes with an equal weight. Positive predicted value **(PPV**) and negative predicted values **(NPV**) are displayed. The figure for the confusion matrix (**CM**) is also displayed. Numbers in square brackets for each statistical feature represent the 95% confidence interval for that measure. **Nan** indicates when a value cannot be computed due to the structure of the confusion matrix.

Supplementary Table S3: LLM performance on combinations of prompts.

Outcome Measure	THR	Model	MPR	MRC	MFS	SP	SN	AUC	ACC	PPV	NPV	CM
MoCA	26	BART	0.791 [0.692–0.880]	0.791 [0.694–0.880]	0.780 [0.679–0.869]	0.870 [0.742–0.971]	0.711 [0.561–0.848]	0.791 [0.694–0.880]	0.783 [0.681–0.870]	0.870 [0.741–0.971]	0.711 [0.558–0.850]	[[27;4]; [11;27]]
		BART	0.785 [0.683–0.879]	0.788 [0.686–0.881]	0.780 [0.678–0.870]	0.838 [0.700–0.960]	0.737 [0.591–0.872]	0.788 [0.686–0.881]	0.783 [0.681–0.870]	0.848 [0.714–0.964]	0.722 [0.568–0.865]	[[26;5]; [10;28]]
		BART	0.785 [0.683–0.879]	0.788 [0.686–0.881]	0.780 [0.678–0.870]	0.838 [0.700–0.960]	0.737 [0.591–0.872]	0.788 [0.686–0.881]	0.783 [0.681–0.870]	0.848 [0.714–0.964]	0.722 [0.568–0.865]	[[26;5]; [10;28]]
	25	GPT2	0.765 [0.661–0.861]	0.768 [0.663–0.865]	0.763 [0.660–0.860]	0.770 [0.632–0.897]	0.766 [0.607–0.906]	0.768 [0.663–0.865]	0.768 [0.667–0.870]	0.719 [0.559–0.870]	0.811 [0.676–0.926]	[[30;9]; [7;23]]
		GPT2	0.760 [0.656–0.856]	0.763 [0.657–0.860]	0.751 [0.643–0.854]	0.692 [0.543–0.833]	0.833 [0.690–0.962]	0.763 [0.657–0.860]	0.754 [0.652–0.855]	0.676 [0.516–0.824]	0.844 [0.704–0.964]	[[27;12]; [5;25]]
		BART	0.750 [0.643–0.849]	0.751 [0.644–0.852]	0.748 [0.639–0.848]	0.770 [0.632–0.897]	0.733 [0.567–0.880]	0.751 [0.644–0.852]	0.754 [0.652–0.855]	0.710 [0.545–0.862]	0.789 [0.650–0.909]	[[30;9]; [8;22]]
	24	GPT2	0.834 [0.711–0.939]	0.785 [0.665–0.899]	0.801 [0.681–0.905]	0.940 [0.865–1.000]	0.631 [0.400–0.850]	0.785 [0.665–0.899]	0.855 [0.768–0.928]	0.798 [0.571–1.000]	0.870 [0.774–0.957]	[[47;3]; [7;12]]
		GPT2	0.808 [0.680–0.919]	0.775 [0.653–0.891]	0.785 [0.661–0.891]	0.920 [0.837–0.981]	0.631 [0.400–0.850]	0.775 [0.653–0.891]	0.840 [0.754–0.928]	0.749 [0.500–0.941]	0.868 [0.769–0.957]	[[46;4]; [7;12]]
		RoB-ERTa	0.877 [0.761–0.957]	0.753 [0.636–0.869]	0.783 [0.649–0.896]	0.980 [0.936–1.000]	0.526 [0.294–0.750]	0.753 [0.636–0.869]	0.855 [0.768–0.928]	0.909 [0.700–1.000]	0.845 [0.745–0.932]	[[49;1]; [9;10]]

	23	BART	0.857 [0.705–0.975]	0.790 [0.648–0.925]	0.812 [0.674–0.929]	0.964 [0.909–1.000]	0.616 [0.333–0.882]	0.790 [0.648–0.925]	0.899 [0.826–0.957]	0.799 [0.500–1.000]	0.916 [0.836–0.982]	[[54;2]; [5;8]]
		GPT2	0.820 [0.664–0.958]	0.781 [0.636–0.917]	0.793 [0.647–0.915]	0.946 [0.879–1.000]	0.616 [0.333–0.882]	0.781 [0.636–0.917]	0.884 [0.797–0.957]	0.726 [0.429–1.000]	0.914 [0.833–0.982]	[[53;3]; [5;8]]
		GPT2	0.820 [0.664–0.958]	0.781 [0.636–0.917]	0.793 [0.647–0.915]	0.946 [0.879–1.000]	0.616 [0.333–0.882]	0.781 [0.636–0.917]	0.884 [0.797–0.957]	0.726 [0.429–1.000]	0.914 [0.833–0.982]	[[53;3]; [5;8]]
	22	BART	0.758 [0.591–0.919]	0.739 [0.578–0.897]	0.741 [0.579–0.883]	0.931 [0.857–0.984]	0.547 [0.231–0.857]	0.739 [0.578–0.897]	0.870 [0.783–0.942]	0.601 [0.273–0.900]	0.916 [0.836–0.983]	[[54;4]; [5;6]]
		BART	0.707 [0.555–0.861]	0.722 [0.559–0.883]	0.707 [0.553–0.849]	0.897 [0.810–0.967]	0.547 [0.231–0.857]	0.722 [0.559–0.883]	0.841 [0.754–0.928]	0.500 [0.200–0.800]	0.913 [0.831–0.982]	[[52;6]; [5;6]]
		BART	0.707 [0.555–0.861]	0.722 [0.559–0.883]	0.707 [0.553–0.849]	0.897 [0.810–0.967]	0.547 [0.231–0.857]	0.722 [0.559–0.883]	0.841 [0.754–0.928]	0.500 [0.200–0.800]	0.913 [0.831–0.982]	[[52;6]; [5;6]]
PhQ-9	10	BART	0.859 [0.735–0.956]	0.785 [0.664–0.900]	0.808 [0.684–0.914]	0.610 [0.375–0.833]	0.960 [0.898–1.000]	0.785 [0.664–0.900]	0.867 [0.779–0.941]	0.872 [0.778–0.949]	0.847 [0.615–1.000]	[[11;7]; [2;48]]
		BART	0.791 [0.675–0.897]	0.819 [0.702–0.922]	0.799 [0.684–0.900]	0.777 [0.562–0.947]	0.860 [0.755–0.945]	0.819 [0.702–0.922]	0.838 [0.750–0.926]	0.915 [0.827–0.980]	0.668 [0.450–0.864]	[[14;4]; [7;43]]
		GPT2	0.828 [0.701–0.938]	0.775 [0.652–0.891]	0.791 [0.664–0.900]	0.610 [0.375–0.833]	0.940 [0.865–1.000]	0.775 [0.652–0.891]	0.853 [0.765–0.926]	0.870 [0.774–0.948]	0.786 [0.545–1.000]	[[11;7]; [3;47]]
	5	BART	0.750 [0.646–0.852]	0.750 [0.646–0.851]	0.747 [0.642–0.851]	0.736 [0.581–0.875]	0.765 [0.613–0.900]	0.750 [0.646–0.851]	0.750 [0.647–0.853]	0.743 [0.591–0.882]	0.757 [0.600–0.895]	[[25;9]; [8;26]]

		BART	0.735 [0.628–0.838]	0.735 [0.627–0.838]	0.733 [0.626–0.837]	0.736 [0.581–0.875]	0.735 [0.581–0.875]	0.735 [0.627–0.838]	0.735 [0.632–0.838]	0.736 [0.581–0.879]	0.735 [0.579–0.879]	[[25;9]; [9;25]]
		BART	0.735 [0.628–0.838]	0.735 [0.627–0.838]	0.733 [0.626–0.837]	0.736 [0.581–0.875]	0.735 [0.581–0.875]	0.735 [0.627–0.838]	0.735 [0.632–0.838]	0.736 [0.581–0.879]	0.735 [0.579–0.879]	[[25;9]; [9;25]]
GAD-7	10	RoB-ERTa	0.739 [0.570–0.897]	0.694 [0.552–0.839]	0.705 [0.555–0.841]	0.461 [0.182–0.750]	0.927 [0.852–0.983]	0.694 [0.552–0.839]	0.838 [0.750–0.912]	0.879 [0.788–0.952]	0.598 [0.273–0.900]	[[6;7]; [4;51]]
		RoB-ERTa	0.739 [0.570–0.897]	0.694 [0.552–0.839]	0.705 [0.555–0.841]	0.461 [0.182–0.750]	0.927 [0.852–0.983]	0.694 [0.552–0.839]	0.838 [0.750–0.912]	0.879 [0.788–0.952]	0.598 [0.273–0.900]	[[6;7]; [4;51]]
		RoB-ERTa	0.739 [0.570–0.897]	0.694 [0.552–0.839]	0.705 [0.555–0.841]	0.461 [0.182–0.750]	0.927 [0.852–0.983]	0.694 [0.552–0.839]	0.838 [0.750–0.912]	0.879 [0.788–0.952]	0.598 [0.273–0.900]	[[6;7]; [4;51]]
	5	BART	0.758 [0.652–0.857]	0.763 [0.656–0.864]	0.757 [0.649–0.853]	0.751 [0.577–0.905]	0.775 [0.639–0.897]	0.763 [0.656–0.864]	0.765 [0.662–0.868]	0.816 [0.684–0.930]	0.700 [0.529–0.862]	[[21;7]; [9;31]]
		BART	0.758 [0.652–0.857]	0.763 [0.656–0.864]	0.757 [0.649–0.853]	0.751 [0.577–0.905]	0.775 [0.639–0.897]	0.763 [0.656–0.864]	0.765 [0.662–0.868]	0.816 [0.684–0.930]	0.700 [0.529–0.862]	[[21;7]; [9;31]]
		BART	0.753 [0.647–0.852]	0.761 [0.653–0.859]	0.746 [0.632–0.851]	0.822 [0.667–0.958]	0.700 [0.553–0.838]	0.761 [0.653–0.859]	0.750 [0.647–0.853]	0.848 [0.714–0.967]	0.657 [0.500–0.812]	[[23;5]; [12;28]]

Binary classification results with different thresholds (**THR**) on features extracted from all prompts, in terms of Macro-F_1-score (**MFS**) (the main metric), Specificity (**SP**), Sensitivity (**SN**), and area under the curve (**AUC**) using the three text-based foundation models, **GPT2**, Facebook.BART-base (**BART**), and RoBERTA-base (**RoBERTA**). Macro precision (**MPR**) and Macro recall (**MRC**) refers to averaging individual classes with an equal weight. Positive predicted value **(PPV**) and negative predicted values **(NPV**) are displayed. The figure for the confusion matrix (**CM**) is also displayed. Numbers in square brackets for each statistical feature represent the 95% confidence interval for that measure.
